# Gas origin linked to paleo BSR

**DOI:** 10.1038/s41598-021-03371-z

**Published:** 2021-12-14

**Authors:** Iván de la Cruz Vargas-Cordero, Lucia Villar-Muñoz, Umberta Tinivella, Michela Giustiniani, Nathan Bangs, Joaquim P. Bento, Eduardo Contreras-Reyes

**Affiliations:** 1 Istituto Nazionale di Oceanografia e di Geofisica Sperimentale-OGS, Borgo Grotta 42C, Trieste, Italy; 2 Departamento de Geofísica, Facultad de Ciencias Físicas y Matemáticas, Universidad de Chile, Santiago, Chile; 3 Institute for Geophysics, University of Texas at Austin, Austin, TX USA; 4 Escuela de Ciencias del Mar, Pontificia Universidad Católica de Valparaíso, Valparaíso, Chile

**Keywords:** Environmental sciences, Natural hazards, Solid Earth sciences

## Abstract

The Central-South Chile margin is an excellent site to address the changes in the gas hydrate system since the last deglaciation associated with tectonic uplift and great earthquakes. However, the dynamic of the gas hydrate/free gas system along south central Chile is currently not well understood. From geophysical data and modeling analyses, we evaluate gas hydrate/free gas concentrations along a seismic line, derive geothermal gradients, and model past positions of the Bottom Simulating Reflector (BSR; until 13,000 years BP). The results reveal high hydrate/free gas concentrations and local geothermal gradient anomalies related to fluid migration through faults linked to seafloor mud volcanoes. The BSR-derived geothermal gradient, the base of free gas layers, BSR distribution and models of the paleo-BSR form a basis to evaluate the origin of the gas. If paleo-BSR coincides with the base of the free gas, the gas presence can be related to the gas hydrate dissociation due to climate change and geological evolution. Only if the base of free gas reflector is deeper than the paleo-BSR, a deeper gas supply can be invoked.

## Introduction

Methane is a powerful greenhouse gas that is 30 times more effective than CO_2_ in trapping heat within Earth’s atmosphere in a scale of 100 years^1^. Gas hydrate (GH), present within the sediments of many continental margins on Earth^2^, is an ice-like crystalline compound of water and gas molecules (mainly methane). This natural resource has worldwide distribution^3–5^, containing twice the total carbon of all fossil fuels combined and about one-sixth of all methane on Earth^6^. So, if the GH is destabilized, a massive amount of methane could be released, impacting the global carbon cycle, ocean chemistry, and interactions between the geosphere and the ocean–atmosphere system^7,8^. Furthermore, free gas (FG) is usually present below the GH reservoir, and it can have a significant thickness, locally up to 200 m^9,10^. The base of the GH reservoir and the FG are commonly detected with seismic data as reflections that lie several hundred meters below seafloor, run roughly parallel to it, and are known as the BSR and the base of free gas reflector (BGR), respectively^11–14^. Some of the FG can be related to the GH dissociation at the BSR following a change in pressure and temperature (PT) conditions at the BSR, while the rest may accumulate from migration from shallow biogenic, or deeper thermogenic sources. Modelling of transient PT conditions predicts a potential dissociation of GH at the reservoir base with global warming^15,16^. Furthermore, FG can also be produced from hydrates or formed into hydrates following PT changes associated with fluid overpressure, which are common to subduction zone settings^17^ and are evident from seafloor mud volcanoes. The fluid and gas transport associated with mud volcanos is also known to feed GH and gas reservoirs^18^. Released gas due to GH dissociation across the upper continental slope are particularly concerning as geohazards because they can generate submarine landslides and possibly tsunami increasing risks for the coastal regions^19^. Furthermore, active margins are very active earthquake settings, and earthquakes are an important triggering mechanism for disrupting hydrates^8,20,21^.

Even though direct measurements from drilling provide information on in situ GH quantities, direct measurements are often very limited and have a distribution that is too sparse to obtain a regional-scale estimate. For this purpose, seismic inversion analyses from marine multichannel seismic data are a useful tool to detect BSR and BGR (if present) and obtain a quantitative estimate of gas-phase concentrations^22–26^ (GH and FG). For this reason, the BSR along the Chilean margin has been widely studied by several authors^13,21,27–36^.

Along the central-south Chile margin, GHs have been reported in shallow marine sediments across the continental slope using direct and indirect measurements^13,21,27–38^. GH extends across the margin from the deformation front up the slope to the shelf forearc basins, and along the margin from Valparaiso (33° S) to Patagonia (54° S). This tectonic setting is one of the most seismically active regions on the planet and is regularly exposed to earthquakes > Mw 9.0.

Fluids play a key role in the nucleation and rupture propagation of earthquakes in subduction zones ^39,40^, and are a major agent of advective heat transfer from depths to the surface. Nevertheless, direct measurements of the heat transfer are logistically demanding and expensive, requiring drillholes or heat probes. Therefore, using the pressure (P)—temperature (T) relationship of the Gas Hydrate Stability Zone (GHSZ), the geothermal gradients (GG) can be constrained by the depth of the BSR, and thus, the regional variations along the Chilean margin can be derived^21,28,30,33,41–43^.

**Figure 1:**
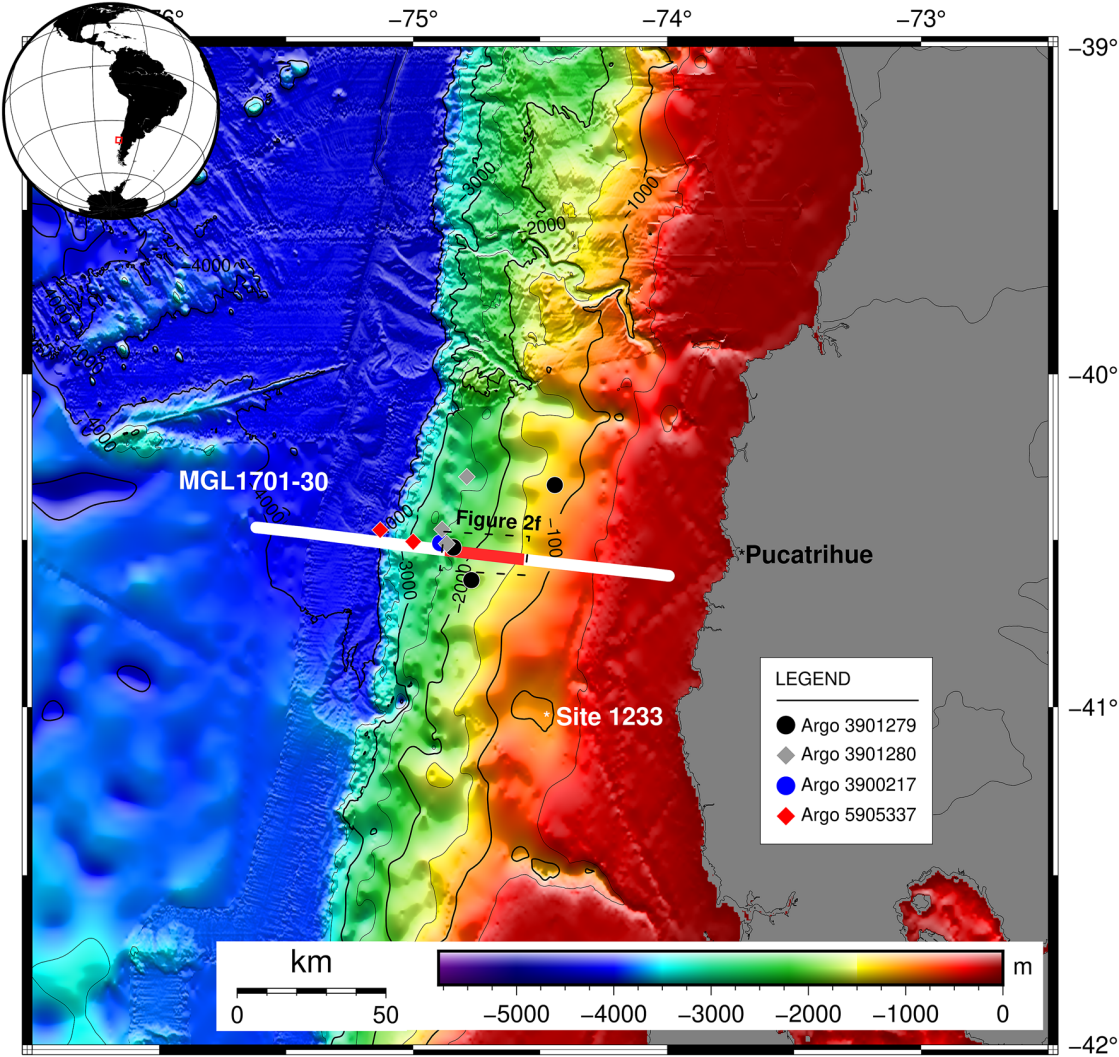
Study area. Location map of the MGL1701-30 seismic and CHIRP lines (white solid line). The red line indicates the analyzed portion of the seismic and CHIRP lines, the dashed black line the location of bathymetry zoom (Fig. 2f) and the white circle is the location of site 1233 drilled by ODP during 2002. The locations of ARGO used for the modeling are reported and explained in legend. The map was generated using GMT (v.6.2.0; https://www.generic-mapping-tools.org/)^49^.

The region offshore Pucatrihue (40° 35′ S) is characterized by the subduction of the Nazca plate beneath the South American plate (Fig. 1). The current convergence rate is about 6.6 mm/year with an oblique azimuth of about 80°^44^. This segment of the Chilean convergent margin is characterized by sediment accretion and subduction^45^, and it forms part of the Mocha Block (the triangulation of the Chilean Trench with the oceanic Valdivia and Mocha Fractures Zones^4^. This region is characterized by the uplift of the continental shelf and forms part of the zone of maximum co-seismic slip (~ 40 m) of the giant 1960 Valdivia earthquake M_w_ 9.5^40,45,46^. Moreover, the area is characterised by a slope sedimentation rate equal to about 2 mm per year^47,48^.

**Figure 2:**
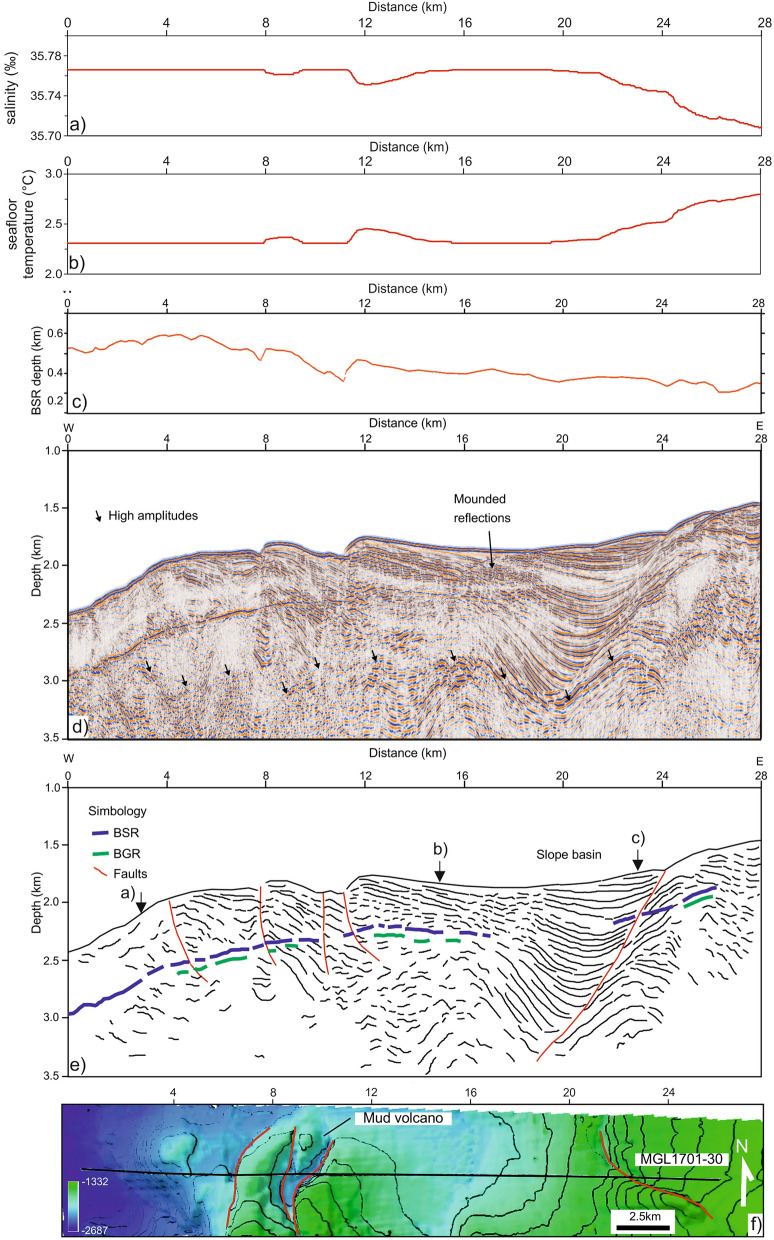
Pre-stack depth migrated and line drawing sections. (**a**) salinity variation along the line; (**b**) seafloor temperature; (**c**) BSR depth profile, (**d**) pre-stack depth migrated section, (**e**) line drawing section and (**f**) bathymetric map across the seismic line. This grid has a size of 103 × 103 m. The black arrows in (**e**) indicate the deeper reflectors, as described in the text. The red lines in (**f**) indicate the main faults. In the sections, the main features are indicated. The map in (**f**) was generated using ArcGIS (v. 10.4.1; www.esri.com).

Sedimentation along the Chilean margin was largely influenced by the Patagonian ice sheet during the Last Glacial Maximum (~ 18,000 years ago), which directly contributed to large sediment volumes, distinctive lithologies and sediment physical properties and indirectly influenced shelf and slope sedimentation from sea level fluctuations during and after the glaciation^46,50^.

Our study is designed to determine the FG migration patterns and compartmentalization of hydrate provinces across the south central Chilean margin by examining the origin of gas in GH/FG systems using a multidisciplinary approach. The offshore Pucatrihue (40° 35′ S; Fig. 1) is an excellent site to study this because this area is characterised by a dynamic geological setting, with frequent large earthquakes and uplift tectonics ^51,52^ and hosts an extensive GH/FG system^36^.

## Data and methods

This multidisciplinary study combines seismic data analysis and theoretical modeling, as described in the following sections.

### Geophysical data

The seismic, CHIRP and bathymetric data were collected in 2017 aboard the *R/V Marcus G. Langseth* during expedition MGL1701 (Lamont Doherty Earth Observatory; LDEO, USA) for the project titled: “Crustal Experiment from Valdivia to Illapel to Characterize Huge Earthquakes (CEVICHE)”^45^. Seismic data is available via repository data “The Marine Geoscience Data System (MGDS)”. We selected the MGL1701-30 seismic line^45^ oriented EW (Fig. 1). The CHIRP data were collected simultaneously with the seismic line and the bathymetric data. In particular, we analyzed 28 km of this line where the BSR presence was recognized. The acquisition parameters are detailed in Table 1.

**Table 1:** Acquisition parameters.

Seismic lines	Source information	Receiver information
MGL1701-30	Volume: 6600 cu in Pressure: 2000 psi Depth: 9 m Shot interval: 50 m Distance to near channel (m): 196	Channels per cable: 1212 Channels recorded: 1212 Group spacing: 12.5 m Cable length: 15,150 m Cable depth: 10 m

The seismic processing was performed by using open-source Seismic Unix software^53^ (SU) and scripts developed ad-hoc^22^. The applied procedure includes: (a) pre-processing and (b) advanced processing. The pre-processing consists of: (a) converting seismic data from segy to SU format, (b) removing noisy traces and direct arrivals, (c) resampling time interval from 0.002 to 0.004 s, and (d) extraction of the first 343 channels (i.e. far offset ~ 4500 m) to avoid effects of the refracted waves found at farther offsets. The advanced processing consists of (a) computing the Kirchhoff Pre-stack Depth Migration (KPSDM) in order to obtain a velocity model, (b) obtaining BSR-derived GG and (c) converting the velocity model into a gas-phase concentration model. This procedure has been tested in several studies^21,30–35^. The velocity and gas phase concentration errors were estimated at < 5% and < 1%, respectively^22,54^.

#### Velocity model building

Continuous reflections (i.e. seafloor, BSR and BGR; Fig. 2) were selected to build a velocity model. The inversion method uses a layer stripping approach^55^, in which each velocity layer is iteratively updated in depth by performing KPSDM algorithm. The velocity analysis is used to calculate semblance of the output of KPSDM (common image gathers; CIGs), from which we determine a residual moveout in depth on the CIGs for velocity corrections (for more details of the method, see^30^). This residual moveout in the semblance corresponds to points of the maximum energy (r-parameter). The optimal migration velocity flattens reflections to a common depth in the CIG; it requires several iterations for the r-parameter value to approach a minimum ~ 0. In this study, the velocity model was generated starting from an initial velocity equal to 1490 m/s (seawater velocity). The selected seismic section, MGL1701-30, was inverted considering 4 main reflectors: seafloor, horizon below it, BSR and BGR. The number of iterations adopted for each velocity layer is reported in Table 2. For the deepest layers (not inverted) a velocity gradient was estimated. Finally, the velocity model was smoothed in order to improve the final image^56^.

**Table 2:** Number of iterations.

Seismic line	Velocity layers			
	Seafloor	Horizon	BSR	BGR
MGL1701-30	3	3	22	9

#### BSR-derived geothermal gradient

The BSR and seafloor depths obtained from the application of advanced processing was indispensable to estimate the GG^32^. The used formula is detailed below:

$${\text{dT}}/{\text{dZ}} = \left( {{\text{T}}_{{{\text{BSR}}}} - {\text{T}}_{{{\text{SEA}}}} } \right)/\left( {{\text{Z}}_{{{\text{BSR}}}} - {\text{Z}}_{{{\text{SEA}}}} } \right)$$

where, T_SEA_ = seafloor temperature extracted from ARGO data^57^ (Fig. 1); T_BSR_ = BSR temperature (gas hydrate stability function as described below^22^); Z_SEA_ = seafloor depth; Z_BSR_ = BSR depth.

Seafloor and BSR depths are extracted from the KPSDM section.

The seawater temperature and salinity were extracted from the ARGO database (see Fig. 1) and averaged in order to obtain a profile of salinity and temperature versus seawater depth. Then, by using the equations of Fofonoff and Millard^58^, the density profile versus depth was calculated. So, the seawater depth was converted to hydrostatic pressure considering a variable density along the seawater column. These profiles were used to extract the values of these properties at the seafloor along the seismic profiles, as reported in Fig. 2. The seabottom temperature ranges from about 2.8 to 2.3 °C moving from the shallower to deeper water, respectively, while the salinity shows a small change (from 34.56 to 34.62‰).

Temperatures at the BSR were estimated from estimated pressures using hydrate/gas stability curves that assumed a pure methane gas composition. We calculated the methane hydrate stability curve by averaging six different methane hydrate phase boundaries with varying effects from salt content^16^. The hydrostatic pressure was calculated from the seafloor to the BSR assuming that the water density is equal to the values obtained at the seafloor from the ARGO data.

#### Gas-phase concentration modeling

**Figure 3:**
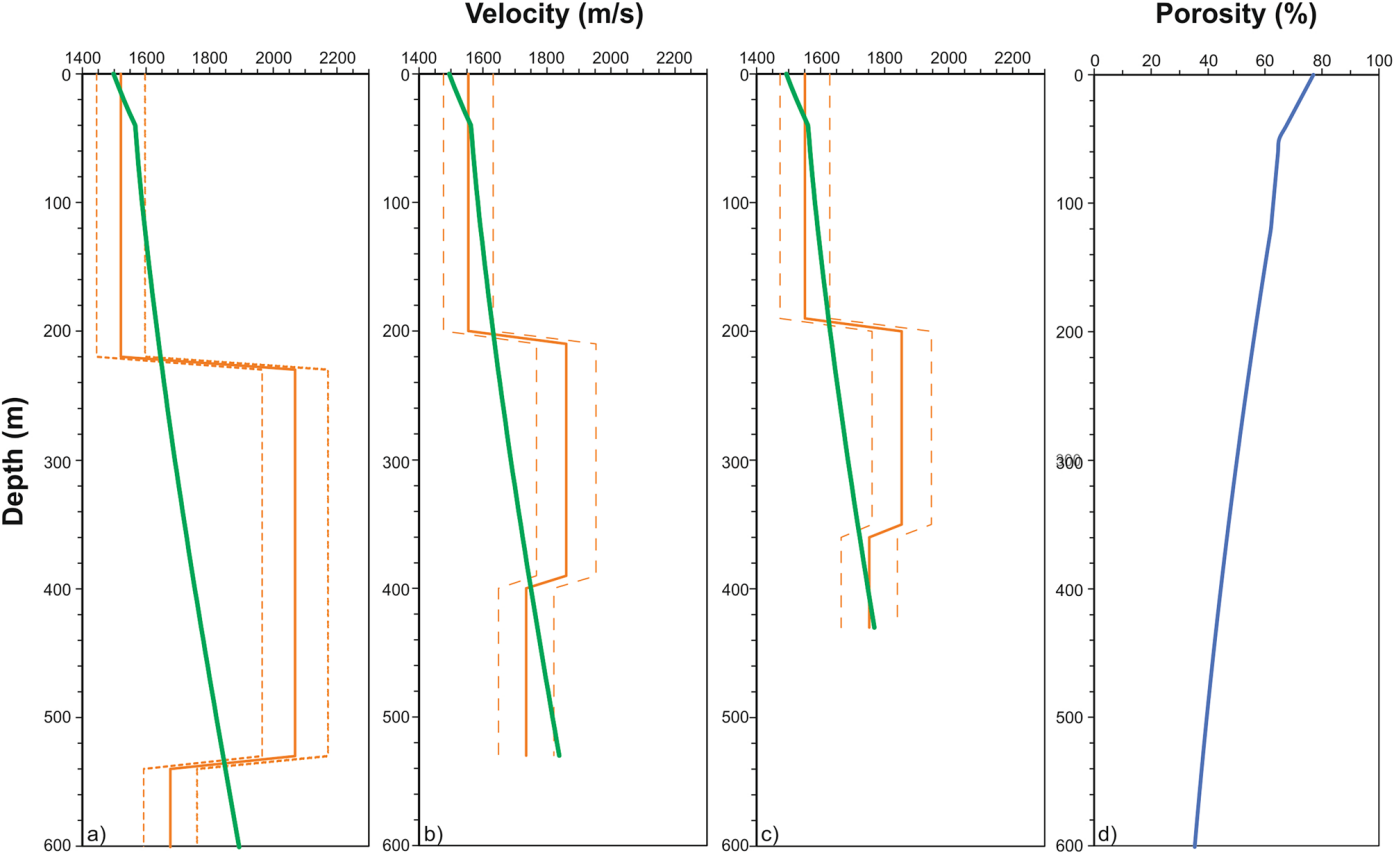
P velocity and porosity. (**a**–**c**) report seismic velocity profiles (orange line) values extracted from seismic velocity model of MGL1701-30 section, and an error of 5% (dashed lines) in the three positions shown in Fig. 2e. The green line represents the background velocity. The (**d**) reports the porosity variations versus depth.

Gas-phase concentration modeling gives information regarding GH and FG amounts. The method compares an inverted velocity model (obtained from KPSDM) with a theoretical model, obtained supposing water only (no free gas) sediments (the so-called background velocity model; Fig. 3a–c). The theoretical model in absence of gas-phase is calculated using the Tinivella method (see details in^59,60^). The comparison of theoretical and inverted velocity models allows us to estimate concentrations of GH and FG. We interpret the positive velocity anomalies as a result of GH, while negative velocity anomalies are attributed to FG. This approach needs to consider two models for the FG distribution: uniform and patchy FG distribution in pore space^59,60^. In the absence of information about the Poisson ratio in the FG layer, we use the velocity distribution as an alternative for determining the appropriate model. In fact, in the case of uniform distribution, very small quantities of FG produce a significant drop in velocity, while a patchy distribution causes a smaller drop in velocity. Figure 3a–c shows the variation of inverted (orange lines) and background velocities (green lines) in the location a, b and c indicated in Fig. 2. For the inverted velocity, we considered an error of 5%, as reported in Fig. 3, (dashed orange lines). For the interval from the seafloor to 120 m depth, we used porosities derived from measurements on drill core samples from ODP Site 1233, located in proximity of our seismic line^47^ (Fig. 1), which were 77%^47^. Figure 3d shows the porosity versus depth used to evaluate the background velocity. The trend of the porosity in depth is consistent with the literature^36^. The final result consists of a gas-phase concentration model in terms of GH and FG in total volume percentage.

### Paleo-BSR modeling

The paleo-BSR has been modeled using the available information on the geologic evolution of the margin since the last glacial maximum time. Using the known current conditions, we model BSR depth changes back in time that correspond to expected changes in sea bottom temperatures and seafloor depths. The initial conditions are the current seafloor bathymetry, the estimated present-day GG along the seismic line, the current sea bottom temperature variable along the seismic line and the methane hydrate stability field, as described above.

The expected changes in P and T are based on known constraints. The sea bottom temperature is considered to be temporally varying^35^: − 3 °C (16,000 years Before Present (BP)), − 2 °C (12,000 years BP), − 1 °C (11,000 years BP). A linear interpolation between these values was applied to model the temperature changes in a continuous record back in time. We also considered the variation of the sea surface salinity versus time as reported by Lamy and Kaiser^61^. The difference between present and past sea surface salinity along the seismic line was propagated versus depth in order to model the seabottom salinity along the seismic line over time. Moreover, temperature and salinity versus time were used to model the seawater density and the hydrostatic pressure along the seismic line over time.

The seafloor depth is affected by several geological phenomena: the sea level change associated with the deglaciation effects (including the glacial isostatic rebound after the last glacial maximum), uplift tectonics, and sedimentation. The sedimentation rate was considered equal to 2 mm/year^48^, while the other phenomena are estimated on the basis of the modeling proposed by Garrett et al.^52^.

We estimated the paleo-BSR and the paleo-seafloor from the second major warming step63, which occurred about 13,000 years BP^62^, in order to determine if the present FG layer is related to past hydrate accumulations.

## Results

### Pre-stack depth migrated section

We identified the following features from the KPSDM seismic imaging (Fig. 2).

**Figure 4:**
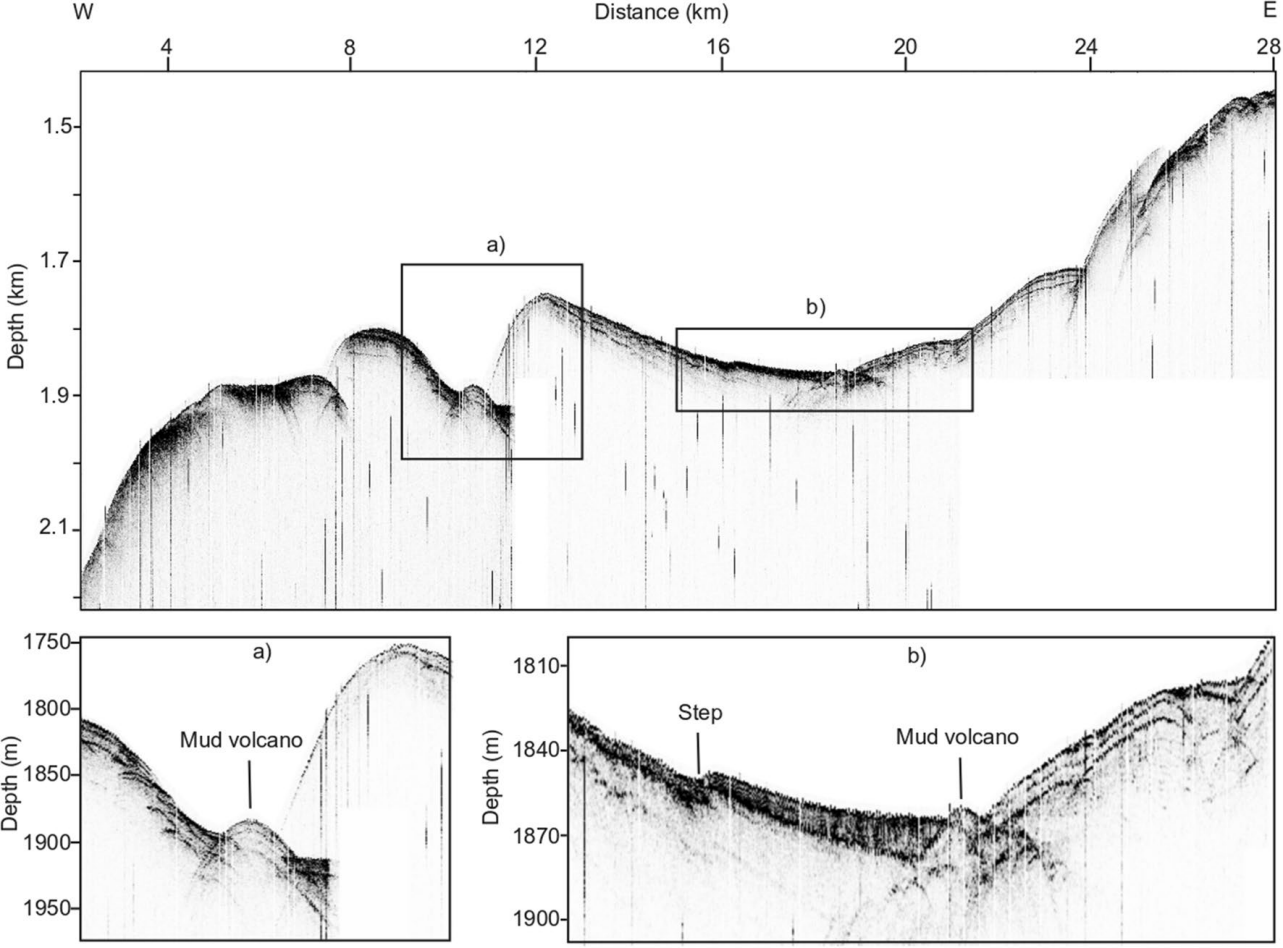
Subbottom profile section. The rectangles in the upper section indicate the position of the zooms (**a**, **b**), in which mud volcanoes were identified.

- In the westernmost part of the line (between 0 and 10 km in Fig. 2d), we identify chaotic and discontinuous reflectors surrounding the BSR, with very low amplitudes. Moving east (between 4 and 10 km in Fig. 2d), these deformed reflectors become more continuous, showing higher amplitudes, and, in the shallowest part (2 km below seafloor), are cut by faults. Here, the seafloor morphology is more complex and, at 4 and 8 km, there are evident features related to faults, as shown in Fig. 2e. In this sector, the BSR is strong and continuous and its depth reaches a maximum of 580 m below seafloor (mbsf; Fig. 2c). Moreover, a weak and discontinuous BGR is recognized below the BSR. Finally, in the deepest part (~ 3 km depth below the surface) we identified discontinuous reflectors with high amplitudes.
- Between 10 and 12 km (Fig. 2d), there is a morphological feature on the seafloor that we interpret as a mud volcano (Figs. 2 and 4). This feature has a length in the NS and WE direction of about 3.3 km and 1.2 km, respectively. Its maximum elevation with respect to the surrounding seafloor is about 60 m. There are two thrust faults flanking this feature that suggests there are compressional stresses that could serve to drive fluid and mud needed to form this feature (Fig. 2f). A similar feature with a smaller dimension, at about 19 km of distance, is also inferred from these data. The height and width are about 6 m and 360 m, respectively, evaluated from CHIRP data (Fig. 4b).
- From 12 to 28 km of distance, in the first 1000 m bsf, we observe subparallel landward dipping and continuous reflectors with high amplitudes. These reflectors show higher amplitudes moving toward the east. From 16 to 19 km of distance, mounded reflections are observed between the seafloor and the BSR. In this portion, the CHIRP data highlight a complex seafloor morphology and an important step of about 4 m at about 16 km along the profile (Fig. 4). From 19 to 24 km distance, divergent reflectors (up to 1.3 km thick) are cut by a main fault with extension of 2 km and seaward vergence (22–24 km in Fig. 2d). Above this fault, the BSR is weak and discontinuous (Fig. 2) until it disappears between 17 and 22 km along the profile. Between 23 and 24 km, it is possible to follow the BSR because it cuts the geological structures. In this sector (12–28 km in Fig. 2d), the depth of the BSR decreases from 500 to 300 m bsf. Note that also the BGR in the section is weak and discontinuous. Below the BSR, it is possible to recognise two zones characterised by low amplitude events and chaotic reflections (3–8 km and 12–17 km in Fig. 3d). Finally, in the deepest part (~ 3 km depth below sea surface) deformed reflectors with high amplitudes are observed (see black arrows Fig. 2d).

### Velocity model

**Figure 5:**
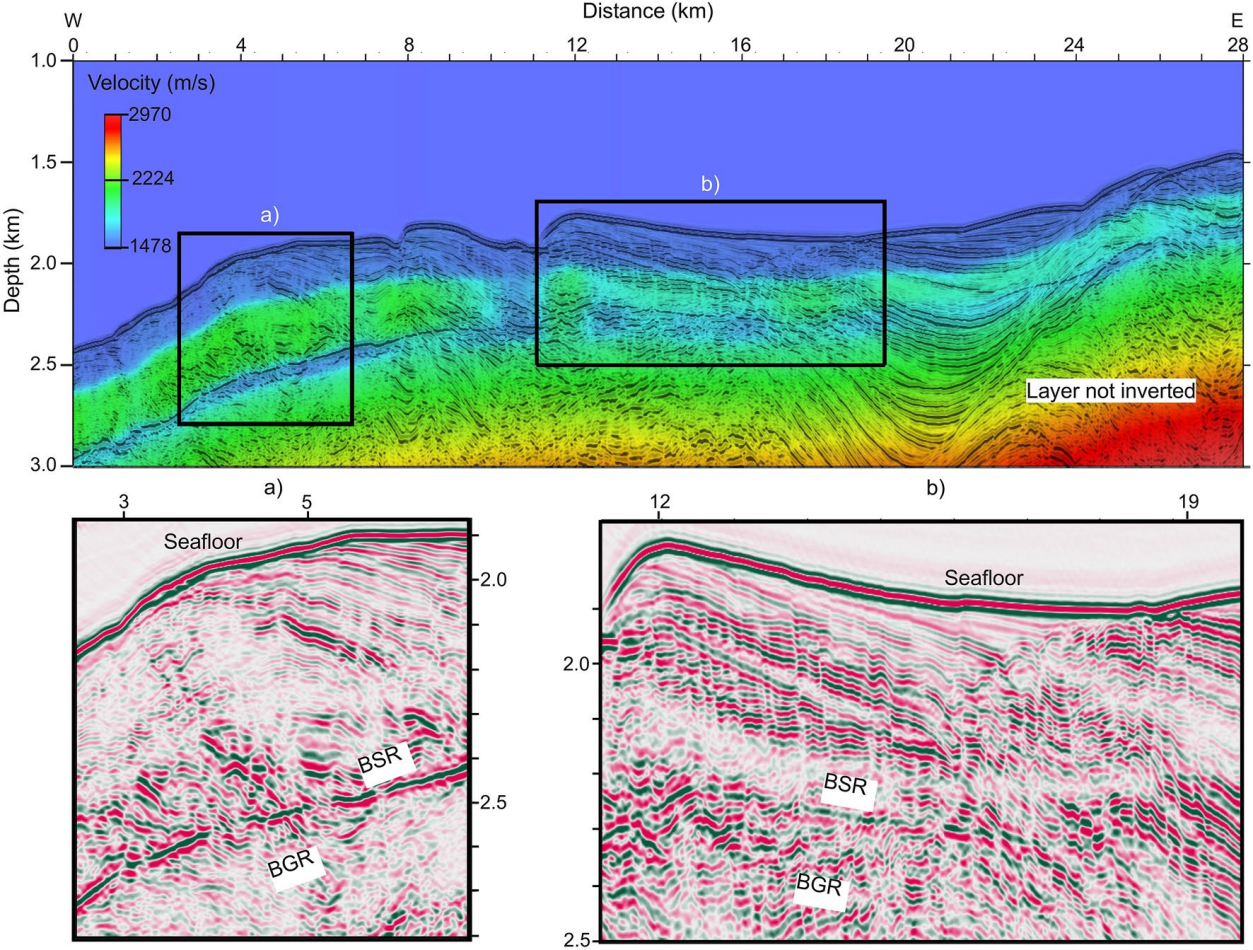
Velocity model superimposed to the KPSDM section. The rectangles in the section indicate the position of the zooms (**a**, **b**), in which BSR and BGR were identified. The figure in (**a**) was generated using Free & Open Source Image Editor GNU Image Manipulation Program GIMP (v. 2.10.24; https://www.gimp.org) and edited-labeled with the Open Source Desktop Publishing Scribus (v. 1.5.6.1; https://www.scribus.net).

The final velocity model consists of three layers (Fig. 5). The first corresponds to a low velocity layer immediately below the seafloor (average velocity of 1550 m/s). The second layer above the BSR shows a high lateral variable velocity (ranging from 1800 to 2100 m/s), and the third layer is characterised by a low velocity below the BSR (ranging from 1600 to 1750 m/s). In the westernmost part, the layer above the BSR shows a consistently high velocity that decreases toward the east. Here, as already mentioned, the BSR is strong and continuous, as shown in Fig. 2d. The third layer is characterised by consistently low velocity, showing lowest values in the western part. To characterize these layers broadly, the lateral velocity distribution above the BSR decreases to the east from 2100 to 1800 m/s, and below it over the same interval velocity increases from 1600 to 1700 m/s (Fig. 5). The thicknesses of high and low velocity layers above and below the BSR reach 250 m and 80 m, respectively. At about 10 km across the profile, in the vicinity of the inferred mud volcano, the three layers show local minima in velocities (less than 2000 m/s).

### BSR derived GG and gas-phase concentrations

**Figure 6:**
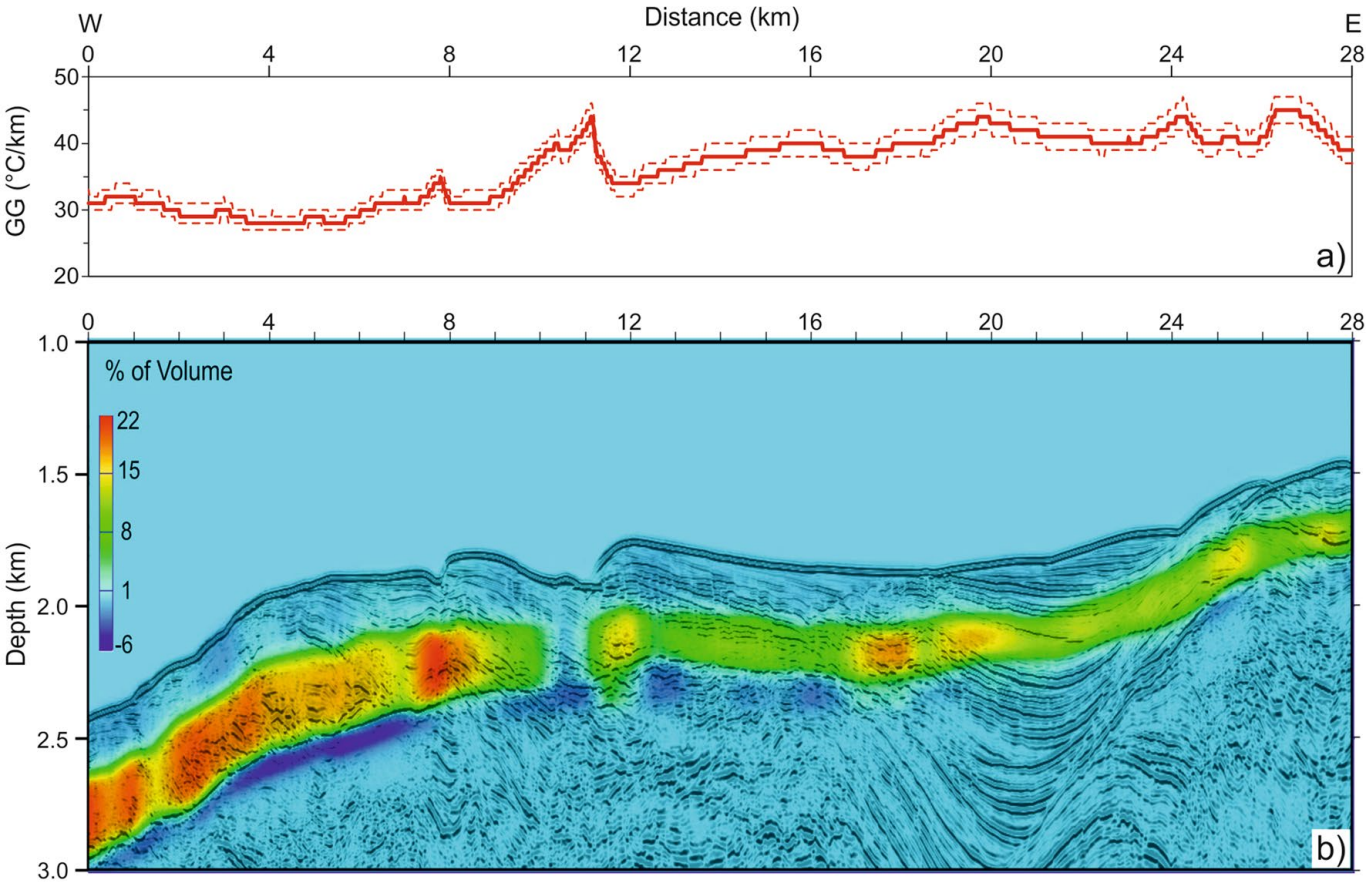
Gas phase’s concentration model. (**a**) BSR-derived GG (red line) with estimated error (dashed red lines); (**b**) gas phase concentration model superimposed on the KPSDM section. Positive values are referred to the GH amount and negative values are referred to the FG amount. The figure in (**b**) was generated using Free & Open Source Image Editor GNU Image Manipulation Program GIMP (v. 2.10.24; https://www.gimp.org) and edited-labeled with the Open Source Desktop Publishing Scribus (v. 1.5.6.1; https://www.scribus.net).

Along the analysed line, the estimated **GG** increases from 30 to 50 °C/km with an average of 40 °C/km from W to E (Fig. 6a). At about 11 km (Fig. 6a), there is a local peak (more than 40 °C/km) compared to the neighboring areas that could be related to fluid circulation, which is consistent with fluid advection associated with formation of the mud volcano and with the locally lower velocities associated with free gas within migrating fluids. The error of the GG is estimated considering the upper and lower limits of the geometrical model extracted from seismic analysis, as reported in Fig. 6a. The error ranges from 1 to 5%. Upwards, both GH and FG concentrations decrease from 22 to 10% and 6–1% of total volume, respectively (Fig. 6b).

### Paleo-BSR

**Figure 7:**
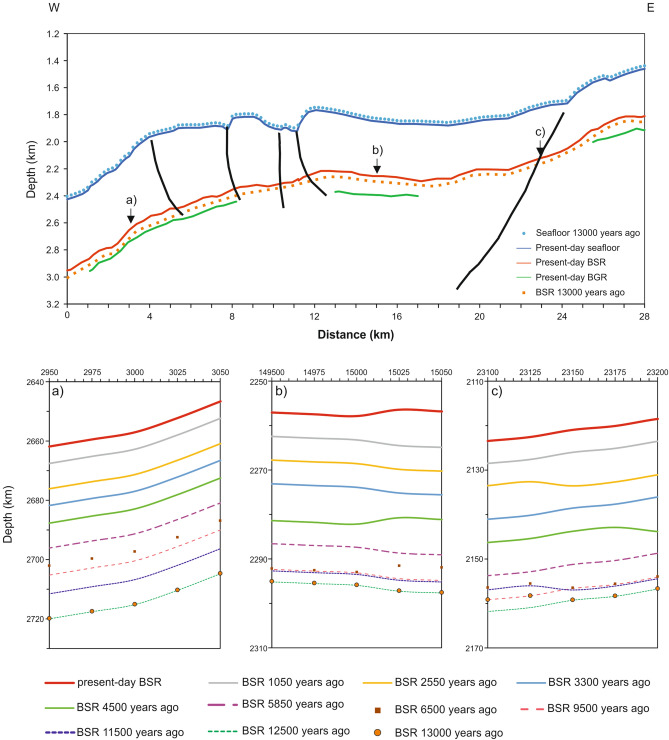
Modeling of MGL1701-30 section over 13,000 years BP. The top panel shows seafloor (blue), BSR (red) and BGR (green) at the present and seafloor (blue dots) and BSR (orange dots) at 13,000 years BP. The black lines represent the faults, reported in Fig. 2. The bottom panels show the BSR depth variation versus time around three selected positions, as indicated by the arrows in the top panel.

On the basis of our modeling, the sea bottom temperature and sea level increased by 2.3 °C and 21 m, respectively, in the last 13,000 years. Our model results (Fig. 7) show that the relationship between the present BSR and the paleo-BSR at 13,000 years BP is quite complex along the line. Figure 7a–c report the variation of paleo-BSR depth versus time at three selected positions. At about 11,000 years BP, paleo-BSR reaches a relative minimum depth because the burial of the BSR from sedimentation exceeds the shallowing of the BSR from other causes. Since 6000 years BP, the water depth change mainly depends on the sedimentation rate. If the maximum error of the GG is considered, the maximum error of the paleo-BSR depth is about 1%.

In the western part of the seismic line (0–10 km; Fig. 7), the depth of the paleo-BSR closely coincides with the present BGR with a maximum difference of about 40 m. Landward, the paleo-BSR depth is shallower with respect to the BGR. Even if the sealevel and the seabottom temperature changes are constant along the seismic line over time, the different behavior of the paleo-BSR depth is clearly related to the variability of the GG; this is because the GH stability curve is not a linear function of temperature with depth.

## Discussion and conclusion

The analysed seismic line shows that this area has large lateral variations in GH and FG across a narrow region (about 30 km). In fact, we can divide the seismic line into two distinctively different parts on the basis of the BSR and BGR characteristics in the seismic profile.

In the western part (between 0 and 10 km), the BSR and BGR are very clear, strong and continuous. In contrast, other reflectors have low relative amplitude. Here, the BSR and BGR layers show higher and lower velocity relative to the regional reference, respectively, which indicates high GH and FG concentrations. Few faults are identified. Moreover, the estimated GG is relatively low (about 30 °C/km).

A structure, interpreted as a mud volcano and delimited by two faults, (between 10 and 12 km) is associated with anomalously low hydrate concentration and relative high GG (highest value about 45 °C/km). This area is characterised by low seismic velocity, where we infer an absence of GH in our models. This mud volcano seems related to compressional tectonics of the area.

In the eastern part, the BSR shows lower amplitudes allowing the clear detection of other reflections. The concentrations of GH and FG are small relative to the seaward portions of the profile, while the GG is higher. Many faults are evident above the GH layer, which could have facilitated fluid flow. Moreover, landward of 18 km, the complex geological features seem due to the presence of deep faults.

As already mentioned, the paleo-BSR shows different behavior along the line. In the western part, it is quite coincident with the BGR, while, in contrast, it becomes shallower reaching the present BSR depth to the east.

In the western part, the distribution of the FG seems mainly controlled by the structural setting that facilitated fluid escape, and thus limited FG accumulation across the BSR. Therefore, the coincidence between present BGR and paleo-BSR suggests that FG could be the result of GH melting after the last glacial maximum. This hypothesis is in agreement with the consideration of some authors who have associated the local presence of BGR with residual paleo-BSR after migration upward of the GHSZ due to global warming and changes in geological settings in an active margin^15,63–66^. In contrast to the western portion of this line, the paleo-BSR is shallower with respect to the BGR along the eastern part, indicating an anomalously thick FG zone for an active margin^65^. This suggests that the gas presence could be related to the GH melting over time, but another sources are possible, too, such as deep supply. Moreover, the evolution of the GH system in the eastern region is impacted by the much higher GG that affects the temporal variation in GH stability. Locally, the sediment stratigraphy can control the gas migration by providing pathways for FG escape to the seafloor, which will in turn control GH distribution. This may have occurred at about 18 km in Figs. 2, 3, 4 and 5. The interpreted mud volcanoes and the complex morphology of the seafloor, both of which are seen in the geophysical data (Figs. 2 and 4), support the fluid circulation. Similar features have been recognised in other areas^66–68^. On the basis of the above considerations, we draw the conclusion that the unexpected thick FG zone in this active margin^65^ is due to a deep gas supply. This is supported by ODP well 1233, located in proximity of our seismic line (see Fig. 1), in which there is a high methane/ethane ratio, indicating a methane origin from methanogenesis of sedimentary organic matter^47^.

**Figure 8:**
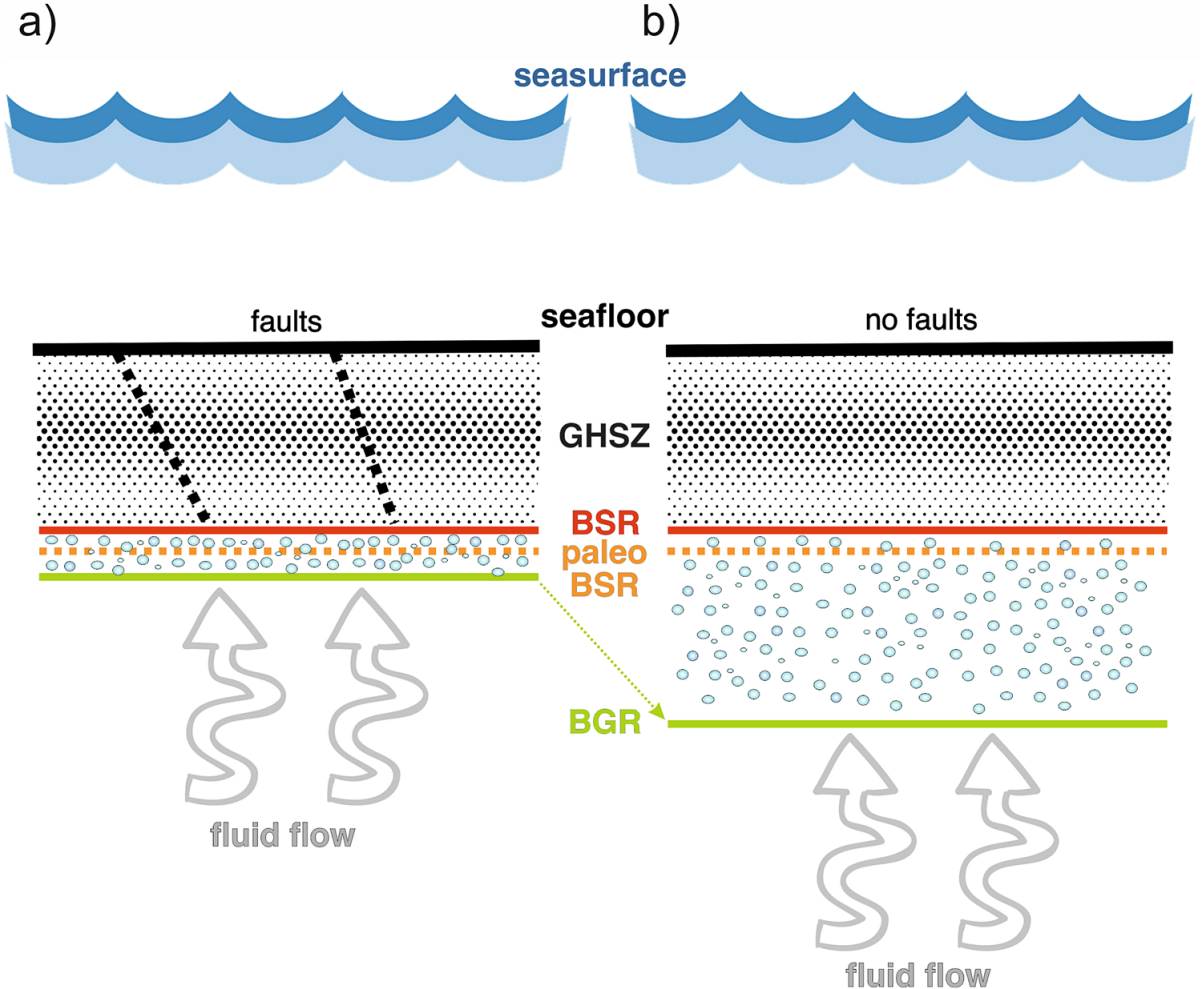
Conceptual plots. The panel shows the position a and b, respectively, reported in this figure. In (**a**) paleo-BSR coincides with the BGR, while in (**b**) BGR is deeper than the paleo-BSR (see text for details). The map was generated using CorelDRAW Graphics Suite 2021 (https://www.coreldraw.com).

On the basis of our study, we hypothesize three different dynamics in the gas hydrate/free gas system, as schematized in Fig. 8. In Fig. 7d, where the paleo-BSR coincides with the BGR, the high FG concentration in a thin layer is related to GH recycling due to climate change and geological evolution after the last glacial maximum. On the other hand, in the areas where the BGR is deeper than the paleo-BSR, supply of gas from a deep source is required to justify the thickness of FG (Fig. 8a). In presence of faults, the negligible thickness of FG is not resolved by seismic data, even if the FG is present due to the BSR detection (Fig. 8b), as already suggested by Haacke et al.^66^.
